# Student experiences and satisfaction with a novel clerkship patient scheduling

**DOI:** 10.1080/10872981.2020.1742963

**Published:** 2020-03-15

**Authors:** Sara Oberhelman, Chris Boswell, Teresa Jensen, Daniel Swartz, Elliot Bruhl, Marcia O’Brien, Kurt Angstman

**Affiliations:** a Department of Family Medicine, Mayo Clinic, Rochester, MN, USA; b Department of Family Medicine, Liberty University College of Osteopathic Medicine, Lynchburg, VA, US; c Southeast Alaska Regional Health Consortium, Juneau, AK, US

**Keywords:** Medical education, primary care family medicine, practice management clerkship

## Abstract

**Background**: Outpatient primary care clerkships are an important part of medical students’ education.Traditional clerkships usually partner a student with a single preceptor in that physician’s clinic. However, it can be quite difficult for the preceptor to balance the educational needs of the students, the expectations of the patients and the organizational demands of the clinic practice.**Objective**: An innovative scheduling model (named “Patients as Teachers” [PAT] clinic) was developed as part of our third-year Family Medicine clerkship. The goal was to increase the students’ opportunities for independence and improve their satisfaction without negatively impacting the flow of the clinic or patient satisfaction.**Design**: The third-year medical students spent part of their clerkship working in the PAT clinic and part of the time working with an individual preceptor in that preceptor’s clinic in the traditional, usual fashion (PAU clinic-precepting as usual). The students completed patient-logs regarding the patients they saw and their level of participation. They also completed a voluntary survey regarding their experiences.**Results**: Students performed more independent interviews (90.3 vs 59.0%) and independent exams (96.2 vs 63.3%) in the PAT clinic than while working with their traditional preceptor (both p<0.01). Students were highly satisfied with the experience with 89.5% stating they would recommend it and 87.7% finding the PAT clinic to be an equal or superior experience to the PAU experience.**Conclusions**: Using a combination of time in the PAT clinic and time with a one on one preceptor in the usual fashion was successful in increasing opportunities for student autonomy and achieving a high level of student satisfaction in our third-year Family Medicine clerkship. Additional opportunities for innovative scheduling could be considered for meeting a variety of clerkship and clinic needs.

## Introduction

Outpatient primary care experiences are essential to a medical student’s education and have been found to increase students’ positive feelings regarding primary care as a future career choice [1,2] while contributing uniquely to students’ medical knowledge [3,4]. Classic models for third-year medical student outpatient clerkships typically involve partnering a student with a physician preceptor to observe and participate in the preceptor’s usual clinic day (‘precepting as usual’ = PAU). This model can create challenges for the preceptor, the student, and the patients. In a PAU model, students are often limited to shadowing and brief opportunities to interview and examine a patient independently due to the time constraints of a full clinic schedule. Attempting to meet the expectations of the patients scheduled in a busy outpatient clinic day while providing a student with the time and opportunities to practice clinical skills can leave preceptors frustrated, rushed, and unsatisfied. This has been described in the literature [5–7] and perceived by our own preceptors.

The frustrations of preceptors led to discussions regarding the ideal structure of outpatient primary care education. The traditional model of education (PAU) has not changed in the last generation, despite the considerable changes in medicine, preceptors, and students. One scenario that eliminated these typical time constraints is the student-led free clinic. Nation-wide, student-led free clinics have been utilized to meet the needs of underserved communities and have received high satisfaction scores from patients and students [8,9]. These models have shown students’ increased ownership of patients’ experiences [8] and improvement in the students’ knowledge and skills [10,11].

Primary care specialties such as Family Medicine require a complex skill set which is difficult to teach by observation in a short time period [6,12]. Innovative scheduling methods (carefully selecting patients for students, having a student sees one patient while the preceptor sees another, multitasking) have been described in traditional PAU models to improve the teaching experience[13], but there are limited data in terms of clinic-wide or clerkship-driven for innovative scheduling.

We developed the Patients as Teachers (PAT) student clinic as a part of our third-year Family Medicine (FM) clerkship in an effort to provide an experience that would increase student autonomy while minimizing any disturbance to patients’ expectations and preceptors’ time constraints. Reports and experiences in student-led free clinics informed our design. Historically, we paired a student with a preceptor in his or her clinic (PAU) for the duration of the students’ clerkship. In the spring of 2014, we implemented a hybrid PAT/PAU clerkship model. The goal of the PAT Clinic was to provide a venue in which students could exercise their developing skills in independent interviews, independent exams and then independently formulate a plan to present to their supervising preceptor; emulating the skills needed to become a successful resident. We hoped also to increase the students’ perceived autonomy while remaining respectful of patients’ time and overall clinic productivity. We hypothesized that students would have more independent experiences while working in the PAT clinic and would express overall satisfaction with the experience.

## Methods

Our FM clerkship is a required three-week outpatient rotation. Our medical school is a small (about 50 students per class), private school affiliated with a large, Midwestern, academic, medical center. There are multiple outpatient primary care practice sites staffed by salaried physicians who serve as preceptors. The PAT Clinic was operated out of a single, suburban practice site with 26,000 Family Medicine, Internal Medicine, and Pediatric patients. Students spent about half their time working in the PAT clinic and the remaining with their PAU preceptor. There was no requirement to participate in the PAU clinics before being assigned to the PAT clinic. We relied on our students’ pre-clinical education to prepare them appropriately for our rotation.

The main goals for the students’ time in the PAT Clinic were to take ownership of patients and develop clinical skills (history taking, examination, developmental of differential diagnosis and formulation and presentation of plan). The main goals for the students’ time in the PAU Clinic were to experience the relationships between patients and family physicians while gaining experience with chronic disease management and preventative care. Three students and one preceptor were assigned to the PAT Clinic each day. Three patients, one per student, were scheduled in each of the two-hour time blocks. The student was expected to take a problem-focused history, perform a pertinent physical exam, develop a differential diagnosis and treatment plan, discuss that plan with the preceptor, discuss the plan with the patient, place orders and write prescriptions, review test results, document a clinical note and all other communications in the electronic medical record, all with appropriate preceptor oversight. Preceptors were required to see each patient, repeat pertinent elements of the history and physical examination, and review and cosign all prescriptions, orders, notes, and results. This ensured patient safety while allowing increased clinical autonomy. Patients were scheduled in a staggered fashion during each time slot which allowed one student to be with a patient, another student to be working on his/her differential diagnosis and plan and the third student to be in consultation with the preceptor.

The PAT Clinic patients were seen for acute appointments and were usually scheduled the day of or the day prior to the appointment, whenever the patient’s primary care provider was not immediately available. The patient was informed that a medical student would be involved in his/her care, the appointment would be longer than usual, and the patient would be an integral part of the student’s educational experience as the student’s primary teacher. Patients were allowed to decline the PAT Clinic appointment slot and would be able to be scheduled with a different provider. Physical or wellness exams, routine prenatal visits, hospital follow-up appointments, and routine chronic disease appointments were excluded. Prior to initiation of this pilot, we asked our Patient Advisory Group to provide input in designing and naming the initiative, consistent with other practice initiatives[14].

At the end of their clerkship, students received a survey by email and the responses were collated and data abstracted by one of the researchers (SSO). The survey asked each student to rate their perceived degree of exposure to several elements of FM (i.e., chronic disease management, office-based procedures, psychiatric care, obstetrical care, etc.), experience regarding the breadth of FM, overall educational experience, proportion of time spent interviewing and examining patients independently and satisfaction with the PAT clinic. PAT and PAU preceptors were encouraged to provide direct and indirect, formative and summative feedback to the medical student during the clerkship. PAU preceptors were required and PAT preceptors were invited to provide written evaluations to the course director for grading purposes.

Each student also completed a log of every patient they saw in the PAT and PAU clinics. They recorded the visit information (date, location), patient’s demographic information (age, gender, reason for visit), and their level of involvement in the encounter (independent interview, independent examination, shared visit with preceptor, shadowing experience). Students were provided with examples of how to classify different experiences and were allowed to include more than one level of involvement for a single patient encounter (i.e., independent interview, shared visit exam).

Statistical analysis of student survey and patient log data from February 2014 through June 2016 was performed with Medcalc software (www.medcalc.org, version 17.4.4). Statistical significance was maintained at a two-tailed p value of <0.05. Due to non-Normal distribution of patient ages, Mann–Whitney testing was utilized for comparison between the groups. Our Institutional Review Board reviewed this project and it was deemed exempt.

## Results

Data were collected from February 2014 through June 2016 (pilot plus the first academic year), 76 students and 1004 patients participated in the PAT/PAU hybrid clerkship model. Fifty-seven students completed the satisfaction survey (response rate 75.0%) and 60 (28 male and 32 female) students’ patient log data were studied.

The patient logs demonstrated an average of 59.0 patient visits during the three-week FM clerkship (total visits = 3538, range of 20–90 patient visits per student). The medical students averaged 15.2 patient visits (total visits = 806, range 6–35 patient visits per student) while in the PAT clinic and 44.7 patient visits (total visits = 2732, range 14–90 patient visits per student) in the PAU clinics over the 3-week clerkship.

The patients seen in the PAT clinic were of a similar age when compared to the PAU patients (Table 1); however, the PAU patients included a higher percentage of children under 2 years of age (5.8% vs. 9.4%, p = 0.002). This is probably due to the well-child visits scheduled during PAU clinic days, which were excluded from the PAT clinic. The overall portion of patients in either the geriatric age range (≥65 years) or pediatric age range (<18 years) was not statistically significantly different between the two groups. The gender of the patients was also similar between the groups. Interestingly, female medical students recorded more visits in the PAT clinic than the PAU clinic (57.5% vs. 53.4%, p = 0.033).

**Table 1.** Demographics of medical student patient encounters in Family Medicine Clerkship, by clinic type

	PAT clinic encounters (N = 805)	PAU clinic encounters (N = 2719)	P =
Patients age: Median (range)	37.0 (0.1–100)	34.0 (0.0–97.0	0.130
Age <2 years old: % (N)	5.8% (47)	9.4% (255)	0.002
Age < 18 years old: % (N)	23.9% (192)	24.9% (678)	0.527
Age ≥ 65 years old: % (N)	13.8% (111)	14.9% (404)	0.448
Female patient: % (N)	56.0% (451)	58.5% (1597)	0.203
Student female gender: % (N)	57.5% (465)	53.4% (1459)	0.033
Independent interview performed: % (N)	90.3% (728)	59.0% (1610)	<0.001
Independent exam performed: % (N)	96.2% (775)	63.3% (1729)	<0.001
Shared visit performed: % (N)	11.4% (92)	27.9% (763)	<0.001
Shadowing only % (N)	0.9% (7)	13.1% (357)	<0.001

Abbreviations: PAT: Patient As Teachers; PAU: Precepting As Usual.

The PAT clinic had an extremely high percentage of visits in which the student performed an independent interview (90.3%) or independent exam (96.2%) which was dramatically increased from the PAU clinic visits with 59.0% of visits with independent interview and 63.3% with independent exam (p < 0.001 for both) (Table 1). Shared visits with the faculty and student were more than twice as common in the PAU clinic, and shadowing experiences were generally only performed in the PAU clinic. Ninety-three percent (N = 53/57) and 91.2% (N = 52/57) of the students indicated that they were allowed to independently interview and independently examine the patient, respectively, a significant proportion of the time while assigned to the PAT clinic.

The students were highly satisfied with the PAT clinic model with 89.5% (N = 51/57) stating they would definitely or probably recommend it. When asked to compare their time between the PAT and PAU models, 35.1% (N = 20/57) felt the PAT was a better experience, whereas only 12.3% (N = 7/57) felt the PAU was a better experience. The remaining 52.6% (N = 30/57) felt neither the PAT clinic nor PAU time was superior to each other. The majority felt the balance between the PAT and PAU clinics was just right (78.9%: 3.3% finding too much time with PAU clinic and 15.8% finding too much time in PAT clinic).

## Discussion

To the authors’ knowledge, no similar clerkship model has been described in the literature. By innovatively scheduling multiple patients to multiple students in longer appointment slots, we were able to provide students the additional time to independently interview and examine a patient. This scheduling did not impact the total number of patients seen daily in our clinic by the preceptor.

Our data demonstrated that this was accomplished with significantly more independent interviews and exams performed in the PAT clinic than PAU. Opportunities for independence and autonomy (with appropriate oversight) are important to a student’s education. It is our hope that engaging students in more resident-physician-like behaviors in the PAT clinic would better prepare our students for their residencies regardless of specialty. Multiple students anonymously provided feedback that their experience in the PAT clinic was the first time they were encouraged to independently formulate, present, and document a plan in the outpatient setting. These students commented that this was a deficiency in their education and that the PAT clinic helped them prepare for the future. Given the time constraints, we did not require directly observed patient encounters in the PAT clinic. We hope to continue to develop the PAT clinic to offer additional experiences such as dedicated observed encounters in the future.

The goals of any medical school clerkship are to impart knowledge and develop clinical skills while also recruiting future physicians to the specialty. The data did show more opportunities for independent interviews/examinations in the PAT clinic, and we hypothesize that this may translate into more opportunities for the development of clinical skills. Since the data show good student satisfaction, we suspect that this could translate into more positive feelings regarding the specialty and future recruitment. Additional studies are needed to examine these hypotheses.

We recognize the value of the traditional PAU educational model. The addition of the PAT element in our clerkship complements the traditional PAU model so that students continue to experience observed and shared visits with their PAU preceptors while ensuring every student also has a guaranteed opportunity to practice interviewing and examining skills independently. As demonstrated by the survey data, the majority of students felt that the balance between the two educational experiences was appropriate. Innovative clerkship models are necessary for the future of medical education.

This study has a number of limitations that could be addressed in the future work. The study took place at a single institution at a single practice site with salaried preceptors, so cross-applicability is limited. The medical student survey was encouraged but voluntary, creating potential response bias. The patient log data were subjective, so one student’s perception of a shared visit may have been another student’s independent experience. However, initial instruction and examples were provided to the students to form a basis for the different levels of interactions.

Since this initial data were obtained, the mixed PAT/PAU model of student education has continued to evolve as part of our clerkship. We have consistently found that patients are satisfied with their experiences. From the student’s standpoint, as long as the schedule is full, the students are happy with the experience as well. Over the course of the past 5 years, we have had the most learning opportunities regarding logistics. Despite limiting the type of visits to acute complaints in the PAT clinic; very complex patients with multiple medical concerns have often been scheduled. With an eye to patient safety and preceptor support, we have decreased the student/preceptor ratio to 2:1. We adapted the schedule so that each student now sees two patients per half day for a total of eight patients each day for a single preceptor. We have experimented with schedules (scheduling two patients at the exact same time versus 15 minute separation) in an attempt to optimize nursing needs and preceptor flow; however, we have not found an ideal scenario since each individual patient/student dyad needs an unpredictable amount of time together.

## Conclusion

Overall, this hybrid model of students spending part of their clerkship time in the PAT clinic working on the development of clinical skills and part of their time in the PAU clinic receiving exposure to the intricacies of FM has been a success. Our students expressed high satisfaction with their increased autonomy and the students had more consistent opportunities for performing independent interviews and examinations.
